# Real-world visual acuity outcomes for patients with naïve neovascular age-related macular degeneration treated with aflibercept, ranibizumab, or bevacizumab in the Republic of Korea

**DOI:** 10.1371/journal.pone.0310381

**Published:** 2024-10-03

**Authors:** Yeo Jin Lee, Seungbum Kang, Jae Yon Won, Young Jung Roh, Ho Ra, Mee yon Lee, Sung Pyo Park, Dong Hyun Jee

**Affiliations:** 1 Department of Ophthalmology and Visual Science, College of Medicine, The Catholic University of Korea, Seoul, Republic of Korea; 2 Department of Ophthalmology and Visual Science, Eunpyeong St. Mary’s Hospital, College of Medicine, The Catholic University of Korea, Seoul, Republic of Korea; 3 Department of Ophthalmology, Saevit Eye Hospital, Goyang-Si, Republic of Korea; 4 Department of Ophthalmology and Visual Science, Yeouido St. Mary’s Hospital, College of Medicine, The Catholic University of Korea, Seoul, Republic of Korea; 5 Department of Ophthalmology and Visual Science, Bucheon St. Mary’s Hospital, College of Medicine, The Catholic University of Korea, Bucheon, Gyeonggi-do, Republic of Korea; 6 Department of Ophthalmology and Visual Science, Uijeongbu St. Mary’s Hospital, College of Medicine, The Catholic University of Korea, Uijeongbu-si, Republic of Korea; 7 Department of Ophthalmology, Kangdong Sacred Heart Hospital, Hallym University College of Medicine, Seoul, Republic of Korea; 8 Department of Ophthalmology and Visual Science, St. Vincent’s Hospital, College of Medicine, The Catholic University of Korea, Suwon, Republic of Korea; Tsukazaki Hospital, JAPAN

## Abstract

**Background:**

To compare the visual outcomes of different anti-vascular endothelial growth factor (VEGF) drugs, including aflibercept, ranibizumab, and bevacizumab, in a real-world setting in Korea.

**Methods:**

We collected data from patients who received monotherapy using one of these three anti-VEGF drugs as naïve treatment after being diagnosed with neovascular age-related macular degeneration. The number of injections and visual acuity (VA) outcomes of each cohort were obtained and pairwise comparisons were performed using propensity score matching.

**Results:**

A total of 254 aflibercept, 238 ranibizumab, and 282 bevacizumab treatment-naïve eyes were included. The mean VA change at 3 years for all cohorts combined was -1.8 letters, and the mean number of injections was 9.4. In the direct comparison of the three drugs, the mean change in the VA letter score was +2.0 letters for aflibercept and -11.7 letters for bevacizumab (P < 0.001). The number of aflibercept injections was significantly higher than the number of bevacizumab injections (P = 0.002). The visual outcomes for aflibercept and ranibizumab were +4.7 letters and -1.9 letters, respectively, and comparable results were obtained (P = 0.13). The VA outcomes for ranibizumab and bevacizumab were also not significantly different (P = 0.09). The numbers of injections for aflibercept, ranibizumab, and bevacizumab were 10.8, 6.7, and 8.8, respectively. Significant differences were observed between the injection frequencies comparisons of aflibercept and ranibizumab and ranibizumab and bevacizumab (P < 0.001 and P = 0.002, respectively).

**Conclusions:**

In the Korean clinical medical environment, which included various confounding factors, especially socioeconomic ones, the aflibercept VA outcome was significantly better than that of bevacizumab, and aflibercept injections were the most numerous. These real-world data imply that the drug effect as well as the environment in which the drug can be sufficiently used affected patient final VA scores.

## Introduction

Age-related macular degeneration (AMD) is one of the main causes of permanent visual impairment and loss in older populations [1,2]. There are two main types of AMD; the atrophic form of dry AMD characterized by gradual loss of central vision due to geographic atrophy; and neovascular AMD (nAMD) associated with increased vascular endothelial growth factor (VEGF) levels and neovascularization, and is responsible for 90% of acute blindness cases. Currently, intravitreal anti-VEGF therapy is the standard nAMD treatment [3].

Aflibercept, ranibizumab, and bevacizumab are the most widely used anti-VEGF agents. All three drugs are generally considered safe and effective for preventing nAMD progression. Several randomized controlled trials (RCTs) and meta-analyses have reported that these three anti-VEGF drugs stabilize or even improve visual acuity (VA) outcomes in patients with nAMD [4].

Studies investigating the impact of these three agents on VA have revealed no significant differences. A comparison of AMD treatment trials (CATT) reported that both ranibizumab and bevacizumab had equivalent effects on VA at 1 year [5] and during a 2-year period [6]. A trial for alternative treatments to inhibit VEGF in age-related choroidal neovascularization (IVAN) [7] also demonstrated that ranibizumab and bevacizumab had similar efficacy. More recently, other RCTs, including the MANTA [8], GEFAL [9], LUCAS [10], and BRAMD [11], confirmed that bevacizumab was not inferior to ranibizumab. Regarding the efficacy of aflibercept and ranibizumab, the VIEW [12] and RIVAL [13] studies showed that they had comparable efficacy in VA outcomes. However, no head-to-head comparative RCT studies have been conducted on aflibercept and bevacizumab. Only an indirect comparison is available from a network meta-analysis, which suggests a mean difference between bevacizumab and aflibercept of 0.02 letters [14]. To date, no randomized prospective comparative trials of these three drugs have been performed. Therefore, the superiority of one anti-VEGF drug over another has not been proven.

Recent emerging real-world studies have allowed us to assess the differences between the results of RCT and population-level treatment outcomes. However, less impressive long-term outcomes have been reported in routine clinical settings. Real-life observations from the AURA study reported that the mean VA gains from baseline to year 1 and 2 were just 2.4 and 0.6 letters, respectively [15]. The SIERRA-AMD study reported -5.2 letters at year 4; the initial VA improvement was not maintained over time, the patients tended to receive fewer injections, and the treatment patterns differed from those in RCTs [16].

RCTs have the advantage of confirming the effectiveness and safety of a drug by minimizing bias. However, the results of RCTs do not represent real-world results because they do not consider the influence of various potential environmental confounding factors. Real-world research reflects what patients actually experience by including various confounding factors, but studies analyzing socioeconomic factors are lacking.

The Fight Retinal Blindness(FRB)! project conducted in Europe has also drawn various conclusions regarding real-world data on AMD. Comparative studies were carried out based on AMD classification or treatment regimens [17,18]. A study from the Netherlands compared the group that started treatment with bevacizumab to the group that started treatment with ranibizumab or aflibercept. The study found no difference in VA, but bevacizumab required more frequent injections and visits, and had a higher rate of drug switching [19]. However, there were no studies that directly compared the two drugs like this one, and particularly none that considered or analyzed the treatment environment(socioeconomic) surrounding the patients.

Therefore, using the Catholic Medical Center Big Data Integration Center, this study compared VA outcomes and injection frequencies of different anti-VEGF drugs, providing a direct comparison of aflibercept, ranibizumab, and bevacizumab from a real-world setting in the Republic of Korea, including socioeconomic factors.

## Material and method

### Information data source

Data from March 2010 to March 2020 were extracted in 2021 from the Clinical Data Warehouse (CDW) of the Catholic University Big Data Integration Center (https://cohort.cmcnu.or.kr). The data extracted for this study were obtained from six university hospital electronic medical record systems from the Catholic University of Korea (Eunpyeong St. Mary’s Hospital, Yeouido St. Mary’s Hospital, Uijeongbu St. Mary’s Hospital, Bucheon St. Mary’s Hospital, St. Vincent’s Hospital, and Incheon St. Mary’s Hospital). Data regarding age, sex, diagnosis, treatment types, anti-VEGF drug types, date of intravitreal injection, date of visit, and best-corrected VA were extracted from the records. Patient identifiers, treatment sites, and clinician data were removed and anonymized.

This study was approved by the Institutional Review Board/Ethics Committee of Catholic Medical Center (Republic of Korea) (2020-2789-0001). Due to the retrospective design of this study and the use of anonymized data, requirements for informed consent were waived by the Institutional Review Board/Ethics Committee of Catholic Medical Center (Republic of Korea).

### Study design

This was a retrospective, comparative, multicenter, non-randomized cohort study of visual outcomes in patients with nAMD treated with aflibercept, ranibizumab, or bevacizumab. Data was used from March 2010 to March 2020, and was extracted in 2021. From the CDW database, treatment-naïve patients (aged >50 years) with a diagnosis code of nAMD (Korean Classification of Disease Code H3135) and a concomitant procedure code for the intravitreal injection of aflibercept, ranibizumab, or bevacizumab were searched. All patients were observed for a minimum of 3 years and received at least one anti-VEGF injection at any time during the study period. The index date was defined as the date of the first anti-VEGF injection. Only patients who received monotherapy with one of the three available anti-VEGF drugs (aflibercept, ranibizumab, or bevacizumab) were eligible for this study. Patients were excluded from participation if they switched to another anti-VEGF agent during the study period or were treated with other treatment options, including photodynamic therapy, steroids, or laser photocoagulation, or if the VA assessment was not accurate. Injections administered for diagnostic purposes or response assessment in cases of drusenoid pigment epithelial detachment or central serous chorioretinopathy were not included. In patients who had both eyes treated with anti-VEGF drugs, the first treated eye was recruited for analysis. The anti-VEGF treatment regimen used to treat patients was not considered because they could not be identified in the CDW system. Using the participant inclusion and exclusion criteria, an overall cohort consisting of all eligible patients was generated. Subsequently, three monotherapy cohorts (aflibercept-, ranibizumab-, and bevacizumab-treated cohorts) were generated from the overall cohort. A baseline imbalance existed among the three cohorts; thus, propensity score matching was used to avoid the risk of bias due to confounding factors. Propensity score-matched cohorts enabled us to perform pairwise comparisons (aflibercept vs bevacizumab, aflibercept vs ranibizumab, and ranibizumab vs bevacizumab).

### Study outcomes

The clinical outcomes were the mean change from VA baseline and the mean number of injections during the 3 years follow-up period in the overall and propensity score-matched cohorts. The final VA change at 3 years was analyzed using pairwise comparisons. All best-corrected VA measurements from the CDW were converted to approximate Early Treatment of Diabetic Retinopathy Study letter scores using previously established guidelines.

### Statistical analysis

The R software (R Core Team, R Foundation for Statistical Computing, Vienna, Austria) was used to perform all statistical analyses. In the unmatched overall cohort, descriptive statistics were calculated using an ANOVA or the Kruskal-Wallis test for continuous variables or the chi-squared test for categorical variables. Case matching was performed using the MatchIt package in R (version 4.2.0; R Core Team, R Foundation for Statistical Computing). The patients were matched based on propensity scores in a 1:1 ratio. The covariates selected for propensity score-matching were age, sex, and baseline VA. The nearest-neighbor matching method without replacement was used. In the propensity score-matched cohorts, continuous variables were compared using the unpaired t-test or Mann-Whitney U test, and categorical variables were compared using the chi-squared test. Statistical significance was defined as P < 0.05. We also compared the cohorts using the standardized mean difference.

## Results

**Fig 1:**
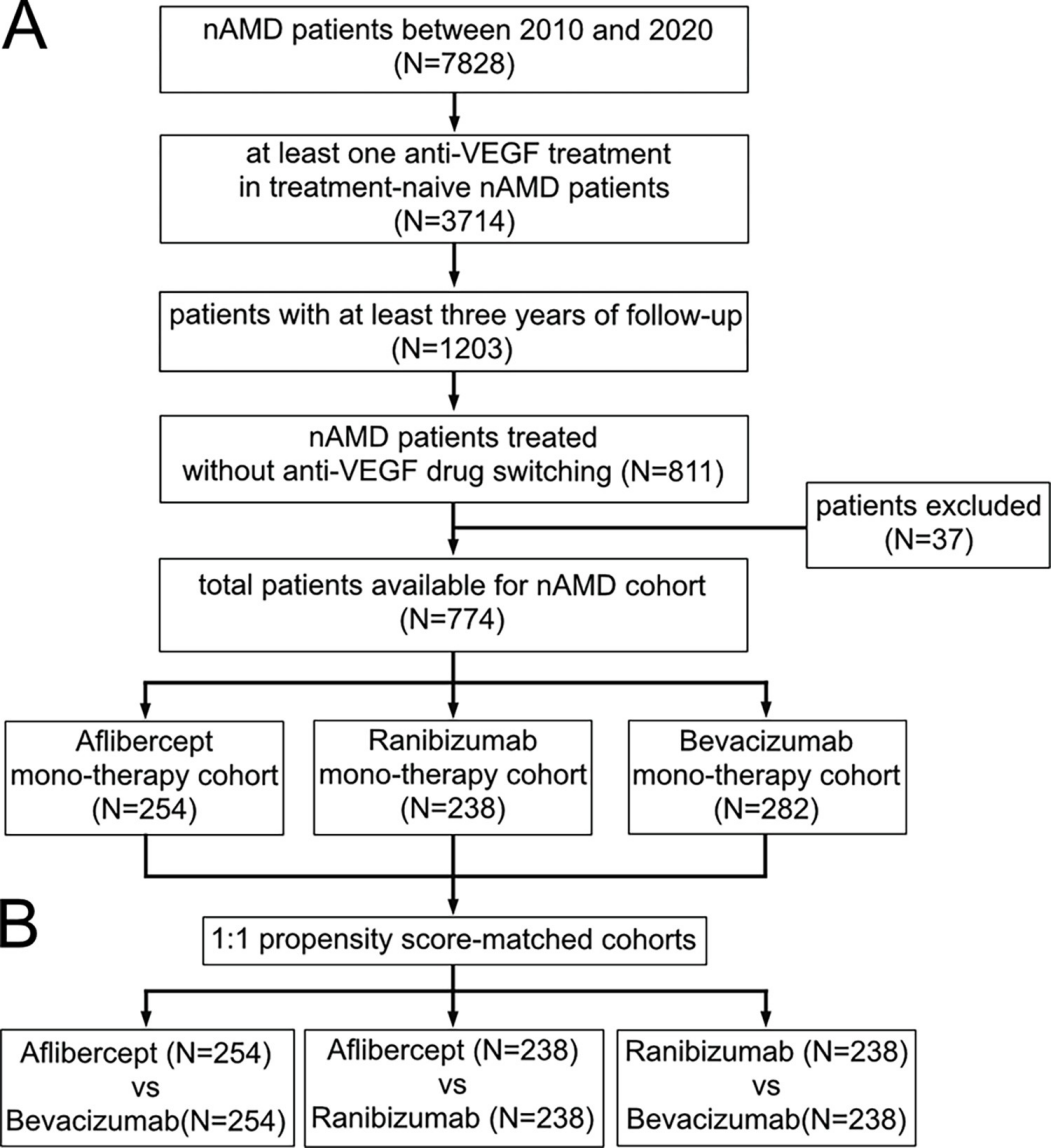
A. Flow charts of the enrolled population B. Propensity score–matched cohort for pairwise comparison.

Based on the inclusion and exclusion criteria, 774 patients were eligible for the study; 254 in the aflibercept cohort, 238 in the ranibizumab cohort, and 282 in the bevacizumab cohort (Fig 1A). Table 1 summarizes the baseline characteristics of all participants. The mean age (SD) for nAMD onset was 68.3 (9.9), 69.1 (8.8), 69.9 (9.0), and 66.3 (10.9) years in the overall, aflibercept, ranibizumab, and bevacizumab cohorts, respectively. The mean baseline VA was 51.5 (23.0), 55.3 (21.5), 48.7 (22.1), and 52.0 (22.5) letters in the overall cohort, aflibercept, ranibizumab, and bevacizumab cohorts, respectively. There were imbalances in the mean age and baseline VA among the three cohorts (P < 0.001 and P = 0.005, respectively). There were significant imbalances in the mean age (aflibercept vs bevacizumab, and ranibizumab vs bevacizumab) and baseline VA (aflibercept vs ranibizumab) with standardized mean difference(SMD) greater than 0.3.

**Table 1:** Baseline characteristics of participants.

Characteristic	Overall	Cohorts (n = 774)						
		Aflibercept (n = 254)	Ranibizumab (n = 238)	Bevacizumab (n = 282)	P-value	SMD (Aflibercept vs Bevacizumab)	SMD (Aflibercept vs Ranibizumab)	SMD (Ranibizumab vs Bevacizumab)
Age, no. (%) 50−59 yr 60−69 yr 70−79 yr 80−89 yr ≥90 yr Mean (SD)	175 (22.6) 219 (28.3) 288 (37.2) 88 (11.4) 4 (0.5) 68.3 (9.9)	41 (16.1) 91 (35.8) 88 (34.7) 33 (13.0) 1 (0.4) 69.1 (8.8)	46 (19.3) 55 (23.1) 109 (45.8) 26 (10.9) 2 (0.8) 69.9 (9.0)	88 (31.2) 73 (25.9) 91 (32.3) 29 (10.3) 1 (0.3) 66.3 (10.9)	<0.001*	0.301	0.026	0.318
Sex, no. (%) female male	371 (47.9) 403 (52.1)	116 (45.7) 138 (54.3)	111 (46.6) 127 (53.4)	144 (51.1) 138 (48.8)		0.089	0.028	0.117
Baseline VA score, no. (%) 65≤ 50−64 35−49 ≤34 mean (SD)	279 (36.1) 218 (28.2) 132 (17.0) 145 (18.7) 51.5 (23.0)	107 (42.1) 82 (32.3) 31 (12.2) 34 (13.4) 55.3 (21.5)	63 (26.5) 67 (28.1) 65 (27.3) 43 (18.1) 48.7 (22.1)	109 (38.6) 69 (24.5) 36 (12.8) 68 (24.1) 52.0 (22.5)	0.005*	0.154	0.324	0.163

VA: Visual acuity, SD: Standard deviation, SMD: Standardized mean difference.

*P-value <0.05.

### Propensity score–matched cohort

Using propensity score matching, the baseline characteristics of the cohorts were well-matched for pairwise comparison. The selected numbers of patients with nAMD were aflibercept (n = 254), bevacizumab (n = 254), aflibercept (n = 238), ranibizumab (n = 238), ranibizumab (n = 238), and bevacizumab (n = 238) (Fig 1B). Baseline patient characteristics of the propensity score-matched cohorts are presented in Table 2. There were no statistically significant differences in mean age and baseline VA between cohorts (P > 0.05, all pairwise comparisons). The SMD for baseline VA between the aflibercept and ranibizumab groups decreased from 0.329 before matching to 0.179 after matching. While this indicates a slight imbalance, it remains below the threshold typically considered to be of concern.

**Table 2:** Baseline patient characteristics of propensity score-matched cohorts.

Characteristic					Propensity score-matched cohorts in each pairwise comparison											
	Aflibercept vs Bevacizumab							Aflibercept vs Ranibizumab					Ranibizumab vs Bevacizumab			
	Aflibercept (n = 254)	Bevacizumab (n = 254)			P-value	SMD		Aflibercept (n = 238)	Ranibizumab (n = 238)	P-value	SMD		Ranibizumab (n = 238)	Bevacizumab (n = 238)	P-value	SMD
Age, no. (%)																
50−59 yr	41(16.1)	65 (25.6)						39 (16.4)	46 (19.3)				46 (19.3)	54 (22.7)		
60−69 yr	91(35.9)	73 (28.7)						80 (33.6)	55 (23.1)				55 (23.1)	72 (30.3)		
70−79 yr	88 (34.6)	86 (33.9)						86 (36.1)	109 (45.8)				109 (45.8)	86 (36.1)		
80−89 yr	33 (13.0)	29 (11.4)						32 (13.5)	26 (10.9)				26 (10.9)	25 (10.5)		
≥90 yr	1 (0.4)	1 (0.4)						1 (0.4)	2 (0.9)				2 (0.9)	1 (0.4)		
Mean (SD)	69.1 (8.8)	68.6 (9.9)			0.278	0.112		69.7 (8.9)	69.9 (9.1)	0.882	0.005		69.9 (9.1)	69.8 (9.0)	0.942	0.002
Sex, no. (%)																
female	116	130						108	111				111	123		
male	138	124			0.213	0.124		130	127	0.782	0.031		127	115	0.271	0.163
Baseline VA score, no. (%)																
65≤	107 (42.1)	104 (40.9)						93 (39.1)	63 (26.5)				63 (26.5)	86 (36.1)		
50−64	82 (32.3)	65 (25.6)						80 (33.6)	67 (28.2)				67 (28.2)	65 (27.3)		
35−49	31 (12.2)	35 (13.8)						31 (13.0)	65 (27.3)				65 (27.3)	36 (15.1)		
≤34	34 (13.4)	50 (19.7)						34 (14.3)	43 (18.0)				43 (18.0)	51 (21.4)		
mean (SD)	55.3 (21.5)	53.8 (21.6)			0.471	0.050		52.0 (21.0)	48.7 (22.1)	0.076	0.179		48.7 (22.1)	50.5 (22.7)	0.299	0.105

VA: Visual acuity, SD: Standard deviation, SMD: Standardized mean difference.

### Visual outcomes and injection frequency in the overall cohorts

**Fig 2:**
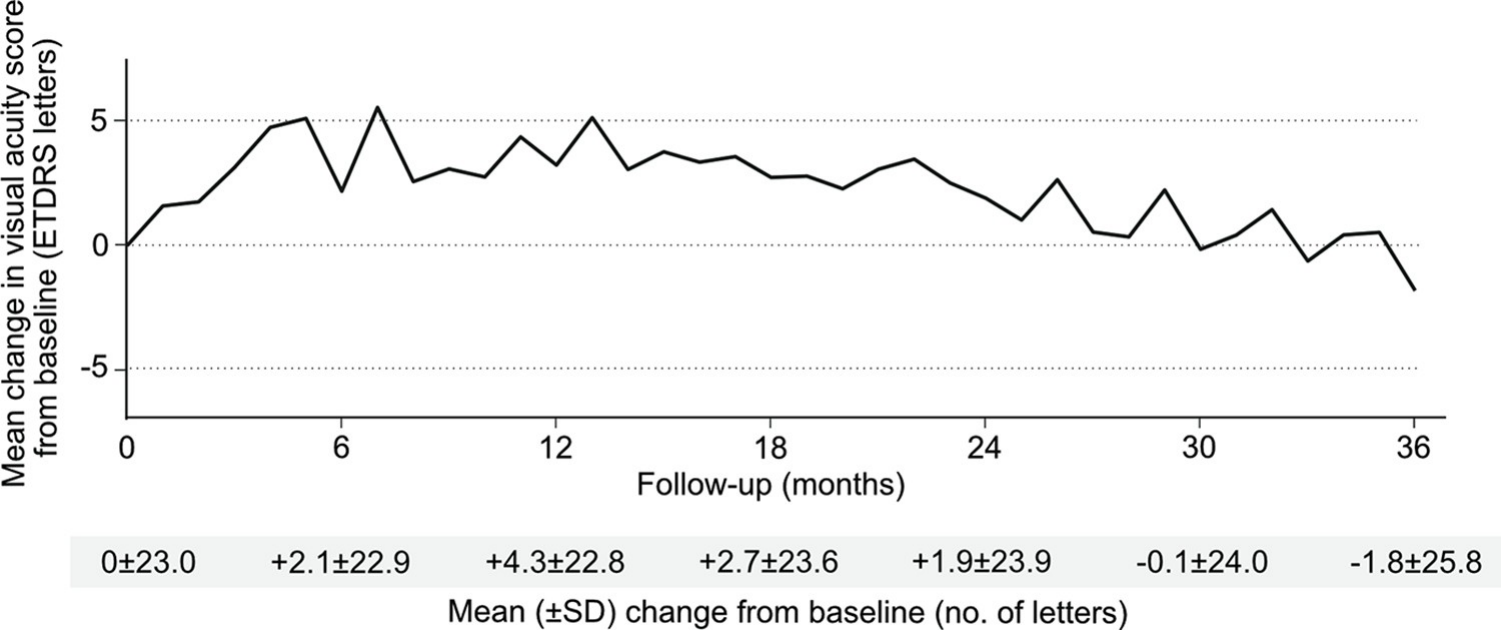
Change in mean visual acuity of all patients.

**Fig 3:**
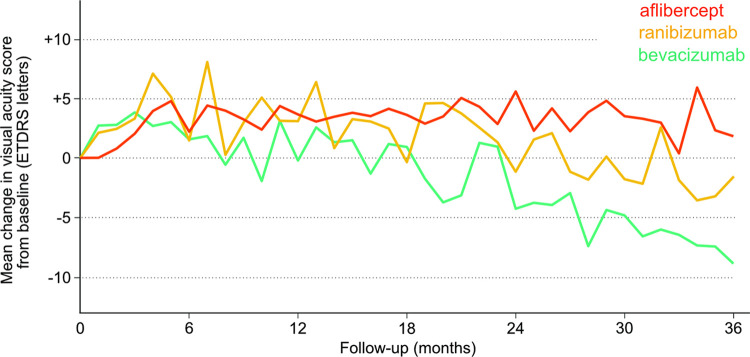
Visual acuity change in each mono-therapy cohort.

The mean VA changes during 3 years for all 744 patients is shown in Fig 2. Most of the improvements occurred during the first 6 months and then started to decline slightly until 3 years. The mean VA changes at 1, 2, and 3 years were +4.3, +1.9, and -1.8 letters, respectively. During the 3 years, the mean (SD) and median numbers of injections administered were 9.4 (5.5) and 7.0, respectively. The VA changes in each monotherapy cohort are shown in Fig 3. The aflibercept cohort had a mean (SD) of 11.3 (6.6) injections received during 3 years and mean VA changes of +3.7, +5.6, and +1.8 letters at 1, 2, and 3 years, respectively. The ranibizumab cohort had a mean (SD) of 6.7 (2.7) injections received during 3 years and mean VA changes of +3.1, -1.2, and -1.4 letters at 1, 2, and 3 years, respectively. The bevacizumab cohort had a mean (SD) of 8.8 (5.2) injections received during 3 years and mean VA changes of -0.2, -4.3, and -8.9 letters at 1, 2, and 3 years, respectively. There was a significant difference in the number of injections among the three cohorts (P < 0.001).

### Visual outcomes and injection frequency in propensity score–matched cohorts

#### Aflibercept vs Bevacizumab

**Fig 4:**
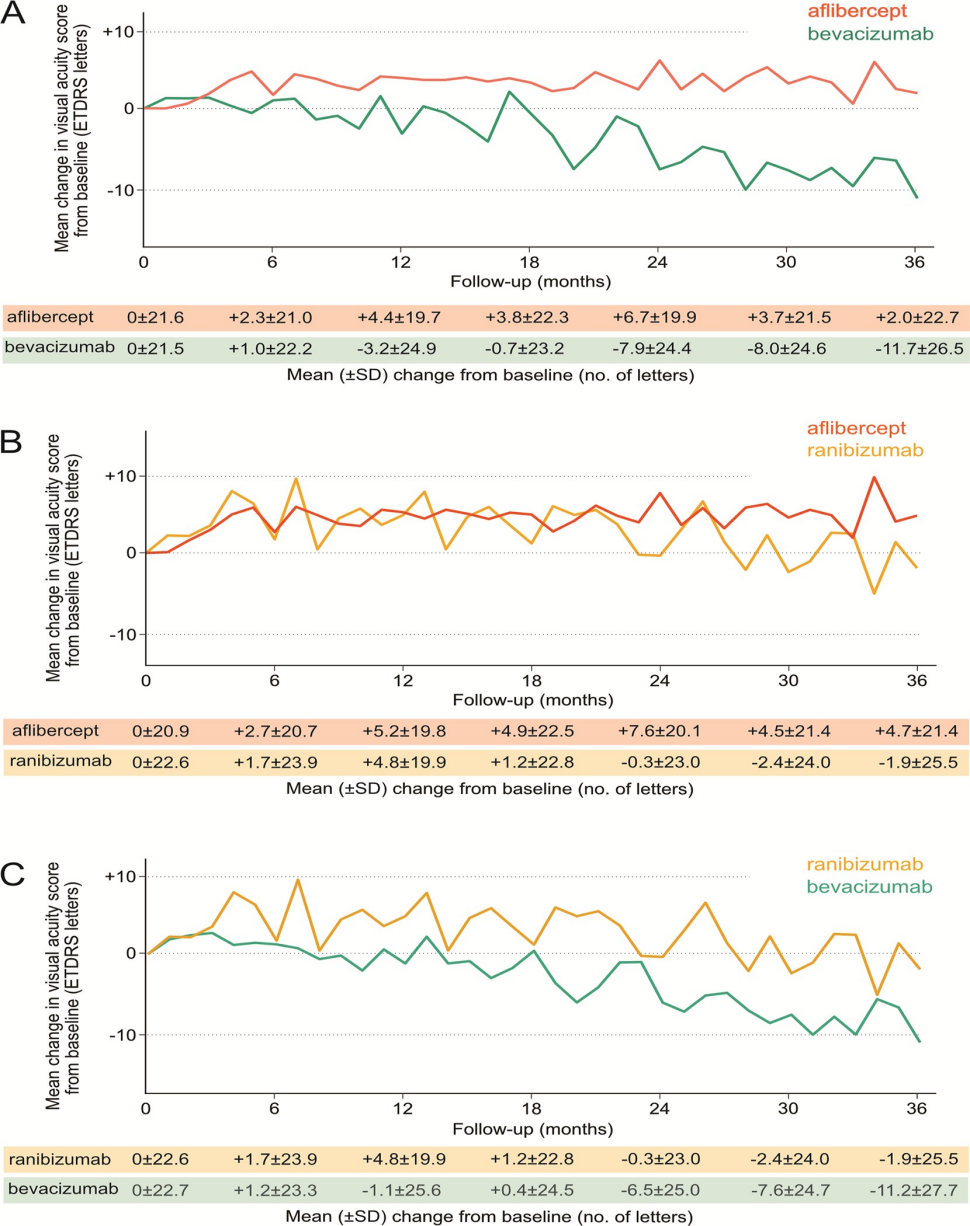
Mean change in visual acuity score from baseline.

**Fig 5:**
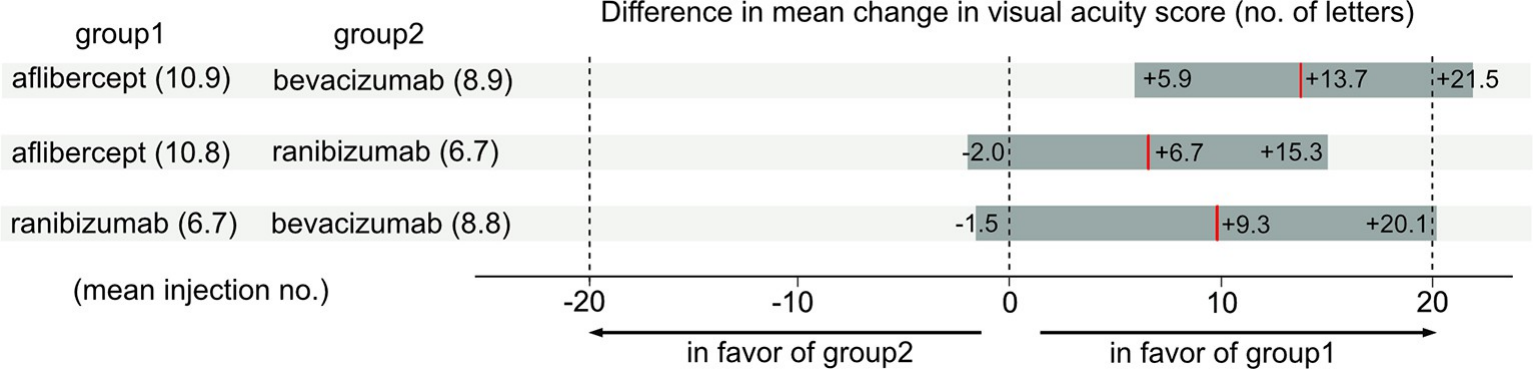
Difference in mean change in visual acuity score.

The aflibercept cohort had a mean (SD) of 10.9 (6.4) injections received and mean VA changes of +4.4, +6.7, and +2.0 letters at 1, 2, and 3 years, respectively. The bevacizumab cohort had a mean (SD) of 8.9 (5.3) injections received and mean VA changes of VA of -3.2, -7.9, and -11.7 letters at 1, 2, and 3 years, respectively (Fig 4). There was a significant difference in the mean VA change from baseline to 3 years between the aflibercept and bevacizumab cohorts (+13.7 letters, 95% confidence interval within +21.5, +5.9) (P < 0.001) (Fig 5). There was a significant difference in the mean number of injections between the two cohorts (P = 0.002).

#### Aflibercept vs Ranibizumab

The aflibercept cohort received a mean (SD) of 10.8 (6.4) injections and had a mean change in VA of +5.2, +7.6, and +4.7 letters at 1, 2, and 3 years, respectively. Ranibizumab cohort had a mean (SD) of 6.7 (2.7) injections and mean VA changes of +4.8, -0.3, and -1.9 letters at 1, 2, and 3 years, respectively (Fig 4). There was no significant difference in the mean VA change from baseline to 3 years between the aflibercept and ranibizumab cohorts (+6.7 letters, 95% confidence interval within +15.3 and -2.0) (P = 0.13) (Fig 5). There was a significant difference in the mean number of injections between the two cohorts (P < 0.001).

#### Ranibizumab vs Bevacizumab

The ranibizumab cohort had a mean (SD) of 6.7 (2.7) injections received and mean VA changes of +4.8, -0.3, and -1.9 letters at 1, 2, and 3 years, respectively. The bevacizumab cohort had a mean (SD) of 8.8 (5.1) injections and showed a mean VA changes of -1.1, -6.5, and -11.2 letters at 1, 2, and 3 years, respectively (Fig 4). There was no significant difference in the mean VA change from baseline to 3 years between the ranibizumab and bevacizumab cohorts (+9.3 letters, 95% confidence interval within +20.1 and -1.5) (P = 0.09) (Fig 5). There was a significant difference in the mean number of injections between the two cohorts (P = 0.002).

## Discussion

This study compared the effectiveness of anti-VEGF drugs on VA in a real-world setting in the Republic of Korea. Compared to bevacizumab, aflibercept had better VA results and required a higher number of injections.

Aflibercept binds to VEGF with a higher affinity than ranibizumab and bevacizumab and binds to placental growth factor. Based on these findings, the advantages of aflibercept over these other two drugs in terms of clinical outcomes have been proposed. Our pairwise analysis revealed that aflibercept outperformed bevacizumab in VA gain. During the 3-year follow-up, the baseline VA was relatively maintained in the aflibercept cohort; however, the mean VA in the bevacizumab cohort continued to decline, resulting in a significant difference of 13.7 letters at the 3-year follow-up. However, the VA outcomes in the aflibercept cohort were not superior to those in the ranibizumab cohort.

To date, few real-world studies have directly compared aflibercept with bevacizumab or ranibizumab with bevacizumab. A study reported that VA outcomes were similar among the three anti-VEGF drugs, in which bevacizumab use was only considered on a compassionate basis in patients with AMD who did not meet the treatment criteria for ranibizumab or aflibercept use [20]. However, several studies in real-world settings have compared the visual outcomes between ranibizumab and aflibercept. Lotery et al. [21] reported 1-year results of mean VA changes were -0.30 letters for ranibizumab and -0.19 letters for aflibercept, confirming ranibizumab is not inferior to aflibercept. Joana et al. [22] also reported there was no difference in VA change between the aflibercept (+2.74 letters) and ranibizumab (-3.07 letters) groups.

The present study also confirmed no differences in VA outcomes between aflibercept and ranibizumab, suggesting that monotherapy with both drugs have equivalent efficacy in real-world settings.

Regarding the comparison of the effects for ranibizumab and bevacizumab, several RCTs have reported similar clinical efficacies of the two drugs, although the results of real-world studies are still unclear. In the present study, the mean VA changes in the bevacizumab cohort continued to decrease during the 3-year follow-up, eventually reaching a difference of 9.3 letters, compared to the ranibizumab cohort, although the difference was not significant. However, a better VA outcome was achieved with fewer injections in ranibizumab cohort (6.7 vs 8.8 average injections in the bevacizumab cohort).

Unlike RCTs, in real-world research, the results must be interpreted considering various variables. These include patient demographics, nAMD types, anti-VEGF agents, treatment regimens, socioeconomic environments, and insurance policies. Regarding nAMD types and treatment regimens, an unknown number of patients with idiopathic polypoidal choroidal vasculopathy (PCV) were possibly included in this study because the PCV frequency in Asia is high, and it is not possible to determine which treatment regimen was used to treat each patient with nAMD by using CDW data alone. This may be one reason for the difference in the number of injections among the three cohorts. Therefore, VA outcomes should be carefully interpreted considering the number of injections.

In our study, the aflibercept cohort had better VA results than the bevacizumab cohort and the number of injections was greater. Since the number of aflibercept injections was high, the VA results of aflibercept seemed better. However, in real-world research, it is important to accept and interpret real-world results as presented rather than prove the efficacy of the drug itself.

Previously, the pro re nata regimen was widely used for anti-VEGF injections, but after the introduction of aflibercept, the treat-and-extend regimen became the main regimen. This may explain the reason the number of aflibercept injections was greater than the number of bevacizumab or ranibizumab injections. However, the most important aspect has been the change in the social security system. The Republic of Korea applies for National Health Insurance (NHI), which is mandated by law and is a universal social insurance that covers the entire Korean population. In the Republic of Korea, the use of bevacizumab is off-label, whereas insurance coverage was implemented in 2009 for ranibizumab and in 2014 for aflibercept. In addition, the number of injections insured was limited to 5 until 2012 and 14 from 2014, and the limit was removed after 2018.

Aflibercept, which is covered by insurance, may have been used more often than bevacizumab because the perception is less expensive, more effective, and has no limit on the number of times it is covered. As the number of aflibercept injections increased, VA also improved. This shows that the social security system plays an important role in the real-world medical environment and patient treatment outcomes. Therefore, efforts must be made to improve the social security system so more people receive more benefits.

This clinical study has several limitations. Some variables in the background characteristics showed marginal significance, which could affect the results of this study. Therefore, to account for these differences in background characteristics, we plan to conduct further research using stricter matching methods, such as using a lower caliper. Risk factors for AMD, such as hypertension, smoking, and cholesterol, could not be identified. However, rather than controlling for these risk factors, we aimed to obtain true real-world data, including all confounding factors. The observation results reflected the actual medical environment and were results experienced by the patients. Another limitation was that the anonymized CDW data did not include information about optical coherence tomography (OCT). However, the final VA was the most important data because the patients ultimately feel the final VA and not OCT results.

The CDW data also has the limitation of not allowing the identification of AMD classifications or treatment patterns that affect prognosis. In Korea, there has not yet been an accurate study on the treatment patterns for AMD, and the treatment patterns vary widely depending on the physician’s preferences and the type of AMD. However, a study confirmed a significant increase in the number of injections after the restriction on the number of reimbursable injections was lifted in 2018 [23]. Before the restriction was lifted, many physicians followed the pro re nata regimen, but it is presumed that after the restriction was removed, more physicians started using treat-and-extend or fixed interval regimens.

Furthermore, This study focuses on monotherapy, which may include patients who respond better to treatment compared to real-world clinical patients, where drug resistance or switching is common. This bias could influence the interpretation of the results. And unfortunately, due to design issues during data extraction from the CDW, it was not possible to extract the total number of cases for each drug. Therefore, we were unable to compare the proportion of patients requiring drug switching or additional treatments for each drug. Further research on these aspects is needed.

Lastly, although Korean insurance covers the entire population, there are exclusion criteria for coverage depending on the AMD lesions. These criteria include cases where ’corrected vision is 0.1 or less, severe scarring, or geographic atrophy’ is present, indicating a poor prognosis, or where ’the lesion is located outside the fovea, or vascular activity such as subretinal fluid or edema is not clearly defined.’ This exclusion can introduce a selection bias, which is a limitation of the study.

This study is meaningful because it compared the degree of vision improvement of anti-VEGF agents, including both socioeconomic factors and the number of injections that affect vision in the real world. Additionally, the strength of our study, compared to other real-world studies, was that the confounding factors in a real-world heterogeneous patient population were reduced as much as possible using baseline VA, sex, and age matching. Moreover, we directly compared the real-world efficacies of aflibercept, ranibizumab, and bevacizumab monotherapies using pairwise comparisons. Thus, we obtained a more accurate real-world comparison of these three drugs.

## Conclusion

In the healthcare environment in the Republic of Korea, which includes various confounding factors, especially socioeconomic factors, the VA outcome of aflibercept was significantly better than that of bevacizumab, and the number of aflibercept injections was the highest.

The VA outcomes of aflibercept vs ranibizumab and ranibizumab vs bevacizumab were similar.

It was confirmed that as the number of injections increased, vision improved, which suggests the importance of establishing a social security system that allows for sufficient use of the medication.

## References

[1] Quillen DA. Common causes of vision loss in elderly patients. American family physician. 1999;60(1):99–108. 10414631

[2] Congdon N O’Colmain B, Klaver C, Klein R, Muñoz B, Friedman DS et al. Causes and prevalence of visual impairment among adults in the United States. Archives of Ophthalmology (Chicago, Ill: 1960). 2004;122(4):477–85. doi: 10.1001/archopht.122.4.477 15078664

[3] Kovach JL, Schwartz SG, Flynn HW, Scott IU. Anti-VEGF treatment strategies for wet AMD. Journal of ophthalmology. 2012;2012. doi: 10.1155/2012/786870 22523653 PMC3317200

[4] Mehta H, Tufail A, Daien V, Lee AY, Nguyen V, Ozturk M, et al. Real-world outcomes in patients with neovascular age-related macular degeneration treated with intravitreal vascular endothelial growth factor inhibitors. Progress in retinal and eye research. 2018;65:127–46. doi: 10.1016/j.preteyeres.2017.12.002 29305324

[5] Martin DF, Maguire MG, Ying G, Grunwald JE, Fine SL, Jaffe GJ. Ranibizumab and bevacizumab for neovascular age-related macular degeneration. The New England journal of medicine. 2011;364(20):1897–908. doi: 10.1056/NEJMoa1102673 21526923 PMC3157322

[6] Group CoA-rMDTTR. Ranibizumab and bevacizumab for treatment of neovascular age-related macular degeneration: two-year results. Ophthalmology. 2012;119(7):1388–98. doi: 10.1016/j.ophtha.2012.03.053 22555112 PMC3389193

[7] Chakravarthy U, Harding SP, Rogers CA, Downes SM, Lotery AJ, Culliford LA, et al. Alternative treatments to inhibit VEGF in age-related choroidal neovascularisation: 2-year findings of the IVAN randomised controlled trial. The Lancet. 2013;382(9900):1258–67. doi: 10.1016/S0140-6736(13)61501-9 23870813

[8] Ehlers JP. The MANTA 1-year results: the anti-VEGF debate continues. British Journal of Ophthalmology. 2013;97(3):248–50. doi: 10.1136/bjophthalmol-2012-302489 23087420

[9] Kodjikian L, Souied EH, Mimoun G, Mauget-Faÿsse M, Behar-Cohen F, Decullier E, et al. Ranibizumab versus bevacizumab for neovascular age-related macular degeneration: results from the GEFAL noninferiority randomized trial. Ophthalmology. 2013;120(11):2300–9. doi: 10.1016/j.ophtha.2013.06.020 23916488

[10] Berg K, Pedersen TR, Sandvik L, Bragadóttir R. Comparison of ranibizumab and bevacizumab for neovascular age-related macular degeneration according to LUCAS treat-and-extend protocol. Ophthalmology. 2015;122(1):146–52. doi: 10.1016/j.ophtha.2014.07.041 25227499

[11] Schauwvlieghe A, Dijkman G, Hooymans J, Verbraak F, Hoyng C, Dijkgraaf M, et al. Comparing the effectiveness of bevacizumab to ranibizumab in patients with exudative age-related macular degeneration. The BRAMD study. PloS one. 2016;11(5):e0153052. doi: 10.1371/journal.pone.0153052 27203434 PMC4874598

[12] Heier JS, Brown DM, Chong V, Korobelnik J-F, Kaiser PK, Nguyen QD, et al. Intravitreal aflibercept (VEGF trap-eye) in wet age-related macular degeneration. Ophthalmology. 2012;119(12):2537–48. doi: 10.1016/j.ophtha.2012.09.006 23084240

[13] Gillies MC, Hunyor AP, Arnold JJ, Guymer RH, Wolf S, Pecheur FL, et al. Macular atrophy in neovascular age-related macular degeneration: a randomized clinical trial comparing ranibizumab and aflibercept (RIVAL study). Ophthalmology. 2020;127(2):198–210. doi: 10.1016/j.ophtha.2019.08.023 31619357

[14] Thomas SM, Lillie E, Lee T, Hamid J, Richter T, Janoudi G, et al. Anti-vascular endothelial growth factor treatment for retinal conditions: a systematic review and meta-analysis. BMJ open. 2019;9(5):e022031. doi: 10.1136/bmjopen-2018-022031 31142516 PMC6549720

[15] Holz FG, Tadayoni R, Beatty S, Berger A, Cereda MG, Cortez R, et al. Multi-country real-life experience of anti-vascular endothelial growth factor therapy for wet age-related macular degeneration. British Journal of Ophthalmology. 2015;99(2):220–6. doi: 10.1136/bjophthalmol-2014-305327 25193672 PMC4316940

[16] Khanani AM, Skelly A, Bezlyak V, Griner R, Torres LR, Sagkriotis A. SIERRA-AMD: a retrospective, real-world evidence study of patients with neovascular age-related macular degeneration in the United States. Ophthalmology Retina. 2020;4(2):122–33. doi: 10.1016/j.oret.2019.09.009 31812631

[17] Teo KYC, Squirrell DM, Nguyen V, Banerjee G, Cohn A, Barthelmes D, et al. A multicountry comparison of real-world management and outcomes of polypoidal choroidal vasculopathy: Fight Retinal Blindness! Cohort. Ophthalmology Retina. 2019;3(3):220–9. 31014698 10.1016/j.oret.2018.11.003

[18] Figueras-Roca M, Parrado-Carrillo A, Nguyen V, Casaroli-Marano RP, Moll-Udina A, Gillies MC, et al. Treat-and-extend versus fixed bimonthly treatment regimens for treatment-naive neovascular age–related macular degeneration: real world data from the Fight Retinal Blindness registry. Graefe’s Archive for Clinical and Experimental Ophthalmology. 2021;259:1463–70. doi: 10.1007/s00417-020-05016-9 33219442

[19] Verbraak FD, Ponsioen DL, Tigchelaar‐Besling OA, Nguyen V, Gillies MC, Barthelmes D, et al. Real‐world treatment outcomes of neovascular age‐related macular degeneration in The Netherlands. Acta ophthalmologica. 2021;99(6):e884–e92. doi: 10.1111/aos.14712 33354933 PMC8519105

[20] Corazza P D’Alterio FM Kabbani J, Alam MMR, Mercuri S, Orlans, et al. Long-term outcomes of intravitreal anti-VEGF therapies in patients affected by neovascular age-related macular degeneration: a real-life study. BMC ophthalmology. 2021;21(1):1–8. 34391401 10.1186/s12886-021-02055-6 PMC8364685

[21] Lotery A, Griner R, Ferreira A, Milnes F, Dugel P. Real-world visual acuity outcomes between ranibizumab and aflibercept in treatment of neovascular AMD in a large US data set. Eye. 2017;31(12):1697–706. doi: 10.1038/eye.2017.143 28731052 PMC5733295

[22] Providência J, Rodrigues TM, Oliveira M, Bernardes J, Marques JP, Murta J, et al. Real-world results of aflibercept versus ranibizumab for the treatment of exudative AMD using a fixed regimen. BioMed research international. 2018;2018. doi: 10.1155/2018/9276580 29984251 PMC6011153

[23] Park YM, Son KJ, Chung EJ, Kim SH. Analyzing Treatment Patterns for Neovascular Age-related Macular Degeneration with Expansion of the Korean Health Insurance Policy. Journal of the Korean Ophthalmological Society. 64(10):913–22.

